# Modeling the Nonlinear Dynamics of Intracellular Signaling Networks

**DOI:** 10.21769/BioProtoc.4089

**Published:** 2021-07-20

**Authors:** Oleksii S. Rukhlenko, Boris N. Kholodenko

**Affiliations:** 1 Systems Biology Ireland, School of Medicine and Medical Science, University College Dublin, Belfield, Dublin 4, Ireland; 2 Conway Institute of Biomolecular & Biomedical Research, University College Dublin, Belfield, Dublin 4, Ireland; 3 Department of Pharmacology, Yale University School of Medicine, New Haven, USA

**Keywords:** Cell signaling, Nonlinear dynamics, Multistability, Oscillations, Bifurcations, Ordinary and partial differential equations

## Abstract

This protocol illustrates a pipeline for modeling the nonlinear behavior of intracellular signaling pathways. At fixed spatial points, nonlinear signaling dynamics are described by ordinary differential equations (ODEs). At constant parameters, these ODEs may have multiple attractors, such as multiple steady states or limit cycles. Standard optimization procedures fine-tune the parameters for the system trajectories localized within the basin of attraction of only one attractor, usually a stable steady state. The suggested protocol samples the parameter space and captures the overall dynamic behavior by analyzing the number and stability of steady states and the shapes of the assembly of nullclines, which are determined as projections of quasi-steady-state trajectories into different 2D spaces of system variables. Our pipeline allows identifying main qualitative features of the model behavior, perform bifurcation analysis, and determine the borders separating the different dynamical regimes within the assembly of 2D parametric planes. Partial differential equation (PDE) systems describing the nonlinear spatiotemporal behavior are derived by coupling fixed point dynamics with species diffusion.

## Background

Here, we present a protocol for computational analysis of the nonlinear dynamic behavior of signaling networks and their transitions between different dynamic regimes. The dynamics of spatially homogenous biochemical systems are described by ordinary differential equations (ODE), whereas the spatiotemporal dynamics are described by partial differential equation (PDE) systems. Knowledge about ODE and PDE is needed as a prerequisite for understanding this protocol. Our computational protocol analyzes the systems’ dynamics at fixed spatial points, and it considers the nonlinear spatiotemporal behavior by coupling fixed point dynamics with species diffusion (Tsyganov *et al.*, 2012; Bolado-Carrancio *et al.*, 2020). A variable $x_{i}$ that is the concentration or activity of each network node *i* depends on the time (*t*) in an ODE system and on both the time and the spatial coordinates in a PDE system. The described protocol operates with the already established interaction topology of the signaling network under study; that is, for each node *i* it is known what nodes activate and/or inhibit it. Mathematically, the network topology is determined by the signed incidence matrix of the ODE system,

**Table:** 

$\frac{dx_{i}}{dt}=f_{i}\left(x_{1},\ldots ,x_{n}\right),\;\;\;i=1,\;2,\;\ldots ,\;n$	(1).

If node *j* activates or inhibits node *i* ($\partial f_{i}/\partial x_{j}>0$ or $\partial f_{i}/\partial x_{j}<0$, respectively), the element $\left(i,\;j\right)$ of the signed incidence matrix equals 1 or -1, respectively, and it equals zero if node *j* does not affect node *i*.

Traditional modeling pipelines for ODE systems use parameter optimization procedures, which rely on the fitting of simulated trajectories to experimental data points, and then study the behavior of a calibrated model (Glover *et al.*, 2000; Maiwald and Timmer, 2008; Kreutz and Timmer, 2009; Penas *et al.*, 2015; Thomas *et al.*, 2016; Degasperi *et al.*, 2017; Mitra *et al.*, 2018 and 2019). These pipelines produce satisfactory results when the system under study has a single stable steady state or when the system trajectories are within the basin of attraction of a stable steady state for bistable or multistable biochemical systems. Recently, parameter optimization procedures have been applied to calibrate oscillatory processes and to search for oscillatory and bistable mass-action networks (Pitt and Banga, 2019; Porubsky and Sauro, 2019). However, for biological systems with multiple attractors – such as steady-state focus nodes, limit cycles, or more complex chaotic structures, which can co-exist at the same parameter values – the use of standard parameter optimization pipelines that fit time-series data might be extremely challenging. Also, systems of higher than 2D dimensions can potentially exhibit chaotic dynamics, typically happening according to one of the following scenarios: (i) period-doubling bifurcations, (ii) a transition to chaos through intermittency, and (iii) chaotization of quasi-periodic dynamics (*i.e.*, through the destruction of multidimensional tori) (Argyris *et al.*, 1993; Kuznetsov, 2012).

Key aspects of the behavior of 2D dynamic systems are determined by the shapes of the nullclines (Tsyganov *et al.*, 2012). Likewise, when a high-dimensional system does not exhibit the chaotic behavior, the assembly of nullclines that are projections of quasi-steady-state (QSS) trajectories into different 2D spaces help us understand the intricate system dynamics. To obtain the QSS trajectories, we change only one variable, whereas all other variables became implicit functions of that variable. Parameter optimization software packages developed for systems biology do not operate with nullclines. A critical feature of our computational pipeline is a combination of intensive sampling of the parameters space, identification of the number and stability of steady states, and the calculation and analysis of nullclines. Only when the key properties of nullclines are determined, the standard fitting software [*e.g.*, BioNetFit (Thomas *et al.*, 2016; Mitra *et al.*, 2019) or PEPSSBI (Degasperi *et al.*, 2017)] is used for fine-tuning of the parameters. After the dynamic regimes of the reduced 2D systems are established, the behavior of the original, high-dimensional system is investigated in the vicinity of attractors found for reduced systems. This pipeline allows developing predictive models of such systems in a semi-automatic regime.

**Figure 1.**
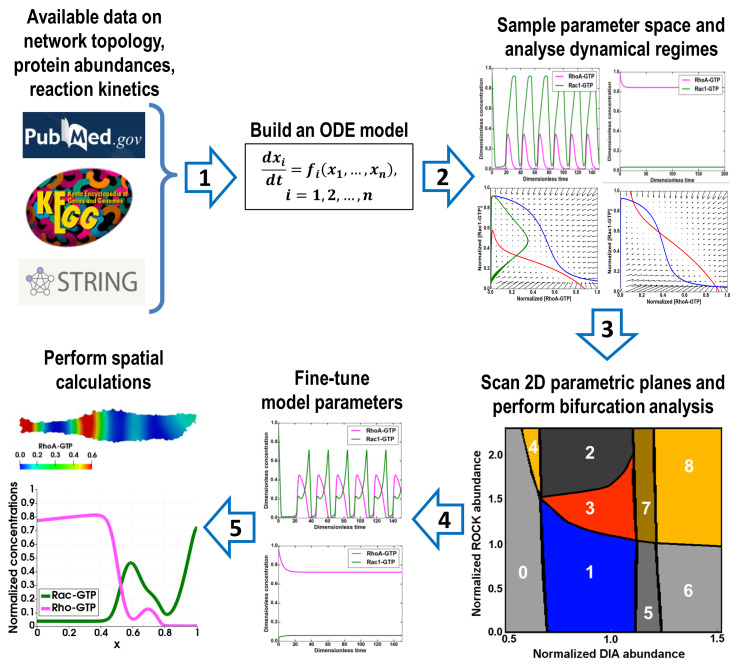
Protocol overview. Consecutive steps of the protocol are indicated by arrows.

A schematic diagram highlighting the main steps of the proposed protocol is shown in Figure 1. The process consists of five successive stages:

1. Build an ODE model of the signaling network and constrain the parameter ranges using the available data on the protein abundances and kinetic constants.

2. Using a software package, *e.g.*, the DYVIPAC package (Nguyen *et al.*, 2015), which uses the libroadrunner library (Somogyi *et al.*, 2015) for importing and solving ODE models, sample the parameter space, determine the number and stability of steady states for a given set of parameters, and analyze the shapes of nullclines by projecting the QSS trajectories into different 2D planes.

3. Scan 2D parametric planes and determine the types of bifurcations that occur in the system.

4. If for given dynamic regimes the system trajectories are measured, fine-tune the parameters to minimize deviations of simulated trajectories from the measured time series data using local search algorithms [*e.g.*, simplex from BioNetFit package (Mitra *et al.*, 2019)].

5. Based on the established dynamic regimes that often differ in distinct spatial regions, formulate a PDE system and perform spatial calculations.

## Equipment

1. Laptop computer
Proposed protocols can be implemented on a laptop computer; however, parameter sampling procedure is time-consuming, and using a computational cluster is advantageous. For example, sampling parameter space of the model (Bolado-Carrancio *et al.*, 2020) to obtain a comprehensive list of different regimes took approximately 5 days at a workstation with 4-core Intel^®^ Core^TM^ i7 Processor. The high-quality scan of 2D parametric plane takes approximately 5 hours given the same computational power. Because the sampling procedures are very well parallelized, the computational time is inversely proportional to the number of available cores.
  Since most computational clusters use Linux-based operating systems, we further refer to software packages mainly developed for Linux.

## Software

1. Python and python libraries, including matplotlib (Hunter, 2007), NumPy (Harris *et al.*, 2020), and scipy (Virtanen *et al.*, 2020)
2. Python version of DYVIPAC software package (Nguyen *et al.*, 2015)
3. Software for parameter fitting of ODE systems, *e.g.*, BioNetFit (Thomas *et al.*, 2016; Mitra *et al.*, 2019), PEPSSBI (Degasperi *et al.*, 2017), *etc.*
4. Software for performing spatiotemporal calculations of reaction-diffusion-convection models, *e.g.*, FiPy (Guyer *et al.*, 2009), OpenFOAM (Jasak, 2009; OpenCFD, 2009) or Virtual Cell (Loew and Schaff, 2001)

## Procedure

1. Build an ODE model of the signaling network based on the available topology of interactions between nodes. Implement the model in a software package that supports export to the SBML format, such as COPASI (Hoops *et al.*, 2006) or BioNetGen (Blinov *et al.*, 2004; Harris *et al.*, 2016).
  - If the kinetics of interactions between two nodes is known, this network connection is modeled mechanistically.
  - For other connections, the interaction kinetics might not yet be exact mechanistically characterized, or these connections operate via intermediate species that are not included in the network under study. These connections are modeled using hyperbolic multipliers $\alpha_{ij}$ that specify the negative or positive influence of the activity ($x_{j}$) of node *j* on the activity (${x54}_{i}$) of node *i* (Tsyganov *et al.*, 2012; Bolado-Carrancio *et al.*, 2020),
    **Table:** 

$\alpha_{ij}=\frac{1+\gamma_{ij}x_{j}/K_{j}}{1+x_{j}/K_{j}}$	(2).
The coefficient $\gamma_{ij}>1$ indicates activation; $\gamma_{ij}<1$ inhibition; and $\gamma_{ij}=1$ denotes the absence of regulatory interactions, in which case the modifying multiplier $\alpha_{ij}$ equals 1. $K_{ij}$ is the activation or inhibition constant.
  - Specify the parameter ranges using the data on the protein abundances and kinetic constants and assign numerical values to model parameters.
  - Non-dimensionalize the model to reduce the number of parameters (Barenblatt, 2003).
  - Export the model to SBML format.
2. Determine dynamical regimes that can be observed in the system.
  - Import the sbml-file of the model into the DYVIPAC package. Sample parameter space of the model using the DYVIPAC package, which determines the number and stability of steady states for a given set of parameters.
  - Rank protein abundances in a model in descending order and pick two proteins (nodes) with the highest abundance. Using the QSS approximation for the other protein activities (Sauro and Kholodenko, 2004; Eilertsen and Schnell, 2020), derive a 2D model from the initial ODE model.
  - Based on the specification of the steady states and the analysis of the shapes of nullclines (Figure 2, for example), determine the dynamic regimes and validate these regimes by the integration of ODEs.
    **Figure 2.**
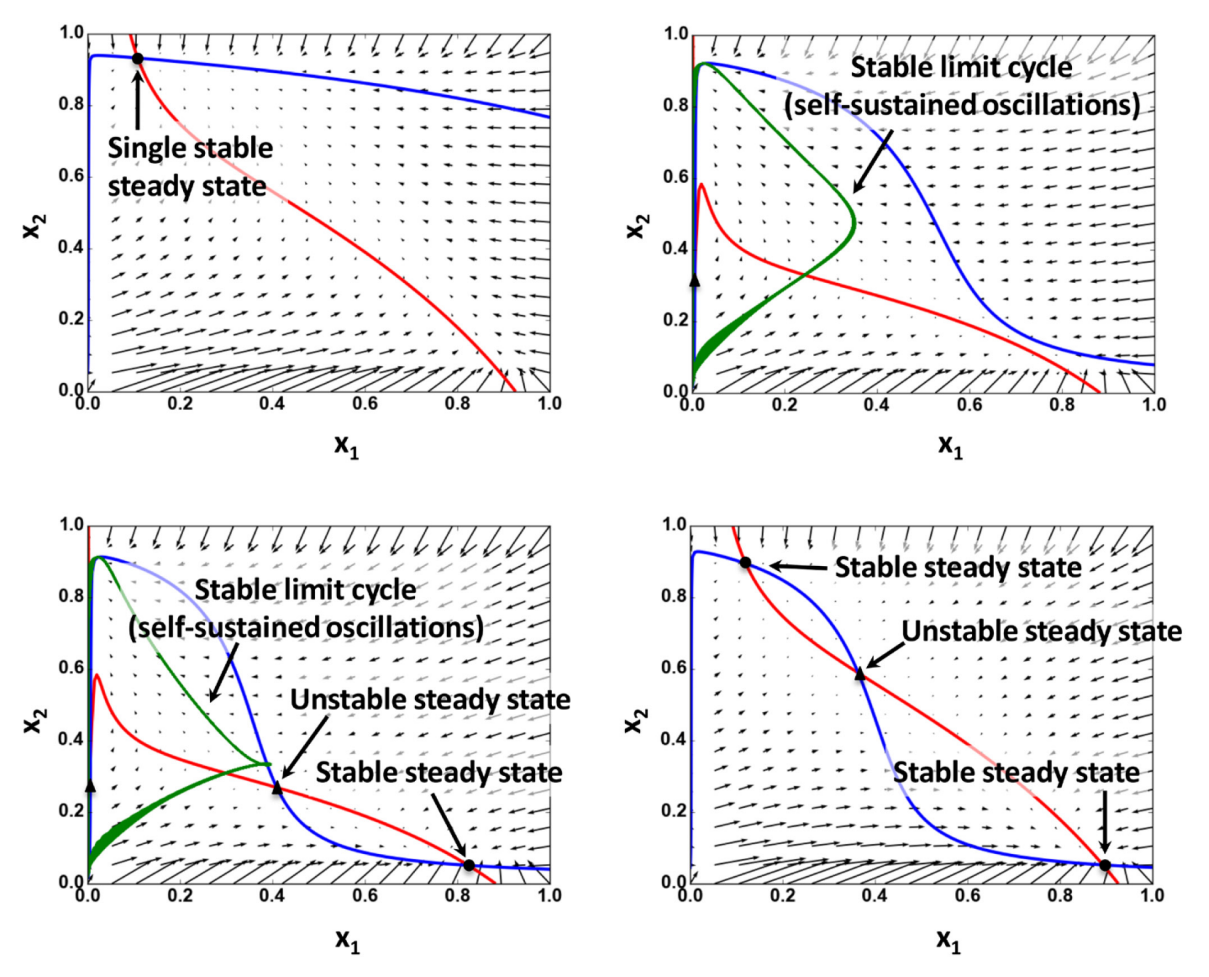
Examples of the nullcline analysis. Nullclines and vector fields calculated for 2D ODE system are derived from a five-dimensional ODE system using a quasi-steady-state approximation. Circles show stable steady states; triangles represent unstable steady states. Red and blue curves are nullclines for variables x_1_ and x_2_, respectively. Green line represents trajectories of limit cycles projected from the original five-dimensional system to 2D space of x_1_ and x_2_ [seeBolado-Carrancio *et al.* (2020) for details].
  - If more than two proteins have high and comparable abundances, make a list of pairs of these proteins and apply the above point c) to the ODE system for each pair.
3. Scan 2D parametric planes and determine the types of bifurcations that can occur in the system.
  - Scan the parameter planes and determine the borders that separate different dynamic regimes (see Figure 3, for example). This step can predict the previously unobserved dynamic regimes, which must be validated in the experiments.
    **Figure 3.**
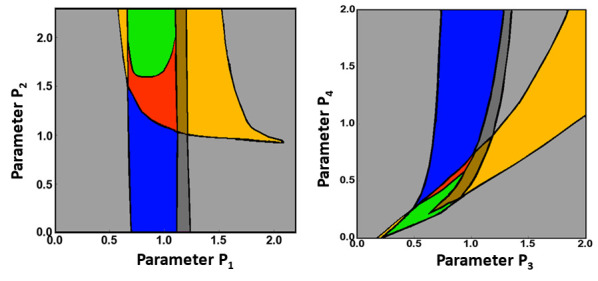
Examples of 2D parametric scans. Different 2D parametric diagrams obtained using scanning of 2-parameter planes. Different colors indicate different dynamical regimes. Black lines represent borders between these regimes where bifurcations happen (see Bolado-Carrancio *et al.*, 2020 for details and Figure 2 there).
  - The analysis of the changes in the number and stability of steady states can reveal local bifurcations, such as the Andronov-Hopf or saddle-node bifurcations (Kuznetsov, 2004). However, non-local bifurcations might exist in a 2D system where a limit cycle can appear or vanish (Nekorkin, 2015). Scan the parameters for dynamic regimes that contain focuses to determine the appearance or annihilation of limit cycles.
4. If there are time-series data, fine-tune model parameters to reproduce the system trajectories for a biologically relevant regime. This step uses standard software packages for parameter optimization (Glover *et al.*, 2000; Maiwald and Timmer, 2008; Kreutz and Timmer, 2009; Penas *et al.*, 2015; Thomas *et al.*, 2016; Degasperi *et al.*, 2017; Mitra *et al.*, 2018 and 2019).
5. Derive a PDE system by adding the terms describing mass transfer processes such as diffusion and convection to the ODE system studied (Tsyganov *et al.*, 2012; Bolado-Carrancio *et al.*, 2020). Perform spatiotemporal simulations using specific software packages, such as OpenFOAM, FiPy, or Virtual Cell.

A set of examples that illustrate the analysis of the dynamics of the RhoA-Rac1 signaling network model and python scripts can be found in “examples.zip” archive.
